# Immunotherapy – 2071. Strail levels decreased after allergen-subcutaneous specific immunotherapy

**DOI:** 10.1186/1939-4551-6-S1-P153

**Published:** 2013-04-23

**Authors:** Arzu Didem Yalcin, Gizem Genc, Saadet Gumuslu, Arzu Didem Yalcin

**Affiliations:** 1 Internal Medicine, Allergy and Immunology, Education and Research Hospital, Turkey; 2 Akdeniz University, Turkey

## Background

TRAIL is present in cells involved in asthma including eosinophils, mast cells, fibroblasts, and airway epithelial cells. It is expressed in airway remodeling and may be linked with the pathways of transforming growth factor-beta1, which is thought to cause damage to the epithelium. The repair process of the epithelium is hindered as a result of increased apoptosis induced by TGF-beta1, which overlaps with the pathways of TRAIL. Analogs of TRAIL could have therapeutical applications for asthma. TRAIL is also seen as the basis for a "miracle" drug for cancer because of its ability to selectively kill cancer cells. Allergic rhinitis is a common health problem affecting the immune system. The homeostasis of the immune system is regulated by apoptosis. In this study, serumcirculating soluble TRAIL levels of allergic rhinoconjunctivitis patients before allergen-specific immunotherapy and after the treatment was evaluated.

## Methods

Subjects with kidney disease, heart disease, liver disease, diabetes mellitus, cancer status, obesity, (body mass index (BMI) ≥30 kg.m2) and autoimmune disease were excluded clinically and serologically. The sTRAIL levels of pre- and post-treated allergic rhinoconjunctivitis patients (n=25) were compared to age and sex-matched healthy individuals (n=25). sTRAIL levels were measured by ELISA. The skin prick test (SPT) were recorded before and after treatment.

## Results

The sTRAIL levels between the pre-treated and control groups were significantly different (p < 0,0001). However, there was no significant difference between post-treated group and healthy individuals (p = 0,801). SPT was a statistically significant difference between the values of the research group before and after immunotherapy (Grasses mixture, Barley mixture, Oleaauropeae, D. Pteronyssinus, D. farinae).

## Conclusions

Allergen- spesific immunotherapy treatment significantly reduced the nasal symptom score across all group I patients studied . In this study, SPT was a statistically significant difference between the values of the research group before and after immunotherapy (Grasses mixture, Barley mixture, Oleaauropeae, D.Pteronyssinus, D. farinae). The sTRAIL levels were decreased after allergen-specific immunotherapy as to healthy individuals’ levels. be a marker of efficacy of immunotherapy in allergic rhinoconjunctivitis patients.

